# Myocardial adaptation after surgical therapy differs for aortic valve stenosis and hypertrophic obstructive cardiomyopathy

**DOI:** 10.1007/s10554-019-01563-3

**Published:** 2019-03-01

**Authors:** Rahana Y. Parbhudayal, Ahmet Güçlü, Alwin Zweerink, P. Stefan Biesbroek, Pierre Croisille, Patrick Clarysse, Michelle Michels, Wim Stooker, Alexander B. A. Vonk, Peter M. van der Ven, Albert C. van Rossum, Jolanda van der Velden, Robin Nijveldt

**Affiliations:** 1 Department of Cardiology, Amsterdam UMC, Vrije Universiteit Amsterdam, Amsterdam Cardiovascular Sciences, De Boelelaan 1117, 1081 HV, Amsterdam, The Netherlands; 2 Department of Physiology, Amsterdam UMC, Vrije Universiteit Amsterdam, Amsterdam Cardiovascular Sciences, Amsterdam, The Netherlands; 3 Department of Cardiology, Isala Klinieken, Zwolle, The Netherlands; 4 Univ Lyon, UJM-Saint-Etienne, INSA, CNRS UMR 5520, Inserm U1206, Creatis, 42023 Sint-Etienne, France; 5 Univ Lyon, INSA-Lyon, Université Claude Bernard Lyon 1, UJM-Saint Etienne, CNRS, Inserm, Creatis UMR 5220, U1206, 69621 Lyon, France; 6 Department of Cardiology, Erasmus Medical Center, Rotterdam, The Netherlands; 7 Department of Cardiothoracic Surgery, Onze Lieve Vrouwe Gasthuis, Amsterdam, The Netherlands; 8 Department of Cardiothoracic Surgery, VU University Medical Center Amsterdam, Amsterdam, The Netherlands; 9 Department of Epidemiology and Biostatistics, VU University Medical Center, Amsterdam, The Netherlands; 10 The Netherlands Heart Institute, Utrecht, The Netherlands

**Keywords:** Aortic valve stenosis, Hypertrophic obstructive cardiomyopathy, Magnetic resonance imaging, Cardiac remodeling

## Abstract

Surgical therapies in aortic valve stenosis (AVS) and hypertrophic obstructive cardiomyopathy (HOCM) aim to relief intraventricular pressure overload and improve clinical outcome. It is currently unknown to what extent myocardial adaptation concurs with restoration of intraventricular pressures, and whether this is similar in both patient groups. The aim of this study was to investigate changes in myocardial adaptation after surgical therapies for AVS and HOCM. Ten AVS and ten HOCM patients were enrolled and underwent cardiac magnetic resonance cine imaging and myocardial tagging prior to, and 4 months after aortic valve replacement (AVR) and septal myectomy, respectively. Global left ventricular (LV) analyses were derived from cine images. Circumferential strain was assessed from myocardial tagging images at the septal and lateral wall of the mid ventricle. Pressure gradients significantly decreased in both AVS and HOCM after surgery (*p* < 0.01), with a concomitant decrease in left atrial volume (*p* < 0.05) suggesting lower diastolic filling pressures. Also, LV volumes, mass and septal wall thickness decreased in both, but to a larger extent in AVS than in HOCM patients. AVR improved wall thickening (*p* < 0.05) and did not change systolic strain rate. Myectomy did not affect wall thickening and reduced septal systolic strain rate (*p* = 0.03). Both AVR and myectomy induced positive structural remodeling in line with a reduction of pressure overload. A concomitant recovery in systolic function however was found in AVR only. The systolic functional deterioration in HOCM patients seems to be inherent to myectomy and the ongoing and irreversible disease.

**Electronic supplementary material:**

The online version of this article (10.1007/s10554-019-01563-3) contains supplementary material, which is available to authorized users.

## Introduction

Left ventricular hypertrophy (LVH) is a common finding in clinical practice and is associated with morbidity and mortality. LVH can be detected in acquired and genetic cardiac diseases. The most common cause for acquired LVH is in aortic valve stenosis (AVS). In response to systolic pressure overload, the myocardium hypertrophies in an attempt to normalize increased wall stress [1]. Patients with severe AVS accompanied with LVH have an increased risk to develop heart failure in the future [2]. Aortic valve replacement (AVR) therapy is recommended in all symptomatic AVS patients, and has been shown to improve left ventricular ejection fraction (LVEF), exercise capacity, and mortality [3]. Considering genetic cardiomyopathies, hypertrophic obstructive cardiomyopathy (HOCM) is the most common cause for LVH with a prevalence ranging from 1:200 to 1:500 [4]. Sarcomeric mutations affect functional properties of the sarcomeres [5] and impair energy metabolism, leading to LVH, most often asymmetric [6, 7]. This asymmetric hypertrophy in combination with systolic anterior motion of the mitral valve can cause left ventricular outflow tract (LVOT) obstruction, leading to heterogeneous symptoms, varying from angina and syncope, to congestive heart failure and sudden cardiac death [8]. The surgical treatment for LVOT obstruction is septal myectomy, which reduces the risk for sudden cardiac death and normalizes left ventricular (LV) pressures [9]. Previous studies have investigated the effect of AVR and septal myectomy on the myocardium separately demonstrating a reduction in intraventricular pressures with subsequent improvement in clinical symptoms and outcome [10–13]. However, it is currently unclear to what extent myocardial structural and functional recovery concurs with restoration of intraventricular pressures, and whether this is comparable in patient groups with similar concentric hypertrophic remodeling, but a different cause (i.e. aortic stenosis vs. genetic). In the current study, we use cardiac magnetic resonance (CMR) imaging to accurately assess structural changes and myocardial function after surgery for AVS and HOCM, and compare this change to a group of healthy controls. We hypothesize that surgical therapies will improve myocardial function in both AVS and HOCM patients.

## Methods

The study protocol was in agreement with the principles outlined in the Declaration of Helsinki and was approved by the Medical Ethics Review committees of the participating hospitals (VU University Medical Center and Onze Lieve Vrouwe Gasthuis in Amsterdam, The Netherlands). All participants gave written informed consent prior to inclusion.

Ten AVS patients eligible for AVR therapy and ten HOCM patients eligible for septal myectomy, were prospectively enrolled in the study between October 2011 and November 2015, as described previously [14]. Inclusion criteria for AVS participants were the presence of isolated AVS with a peak transvalvular pressure gradient of > 50 mmHg, and an aortic valve area < 1 cm^2^, according to the American Society of Echocardiographic guidelines [15]. Inclusion criteria for HOCM participants were LVOT peak pressure gradient > 30 mmHg at rest or during provocation, and presence of clinical symptoms, despite optimal medical treatment. According to the guidelines to undergo surgical intervention in AVS and HOCM patients, different cutoff gradients are advised [16, 17]. The exclusion criteria were any absolute or relative contra-indication for undergoing CMR, the presence of any significant coronary artery disease (> 30%) and a history of hypertension in HOCM patients. LVOT gradient and peak aortic valve pressure gradient were obtained by Doppler echocardiography. Maximal exercise capacity was derived from a cyclo ergometry test when patients reached a point of exhaustion or symptom limitation, prior to, and after surgical therapy with a ramp protocol of 10–20 W min^−1^. To compare myocardial function in AVS and HOCM patients before surgical therapy, 14 gender-matched healthy subjects were included as a control group.

All AVS and HOCM participants underwent a CMR scan, 2 weeks prior to, and 4 months after surgery. CMR was performed on a 1.5 Tesla whole body scanner (Magnetom Sonata or Avanto, Siemens, Erlangen, Germany), using a six-channel phased-array body coil. In all patients and controls, cine images were obtained using a breath-hold segmented k-space balanced steady-state free precession (bSSFP) employing retrospective electrocardiographic gating, with contiguous short axis slices to cover the whole ventricle from base to apex. Ventricular volumes at end-diastole and end-systole, and mass were obtained from the cine short axis images. Left atrial (LA) volumes and emptying fraction (LAEF) were obtained from a stack of transversely oriented slices on a two-chamber view at the level of the lower leading edge of the mitral valve annulus to cover the left atrium [18]. A multiple breath-hold, retrospectively triggered bSSFP myocardial sinusoidal complementary tagged (CSPAMM) images were acquired to create non-invasive markers (tags) within the myocardium [19]. A midventricular short axis plane was positioned at 50% of the distance between the mitral valve annulus and the endocardial border of the apex. Additional details about the CMR acquisition are provided in the Data Supplement.

**Fig. 1:**
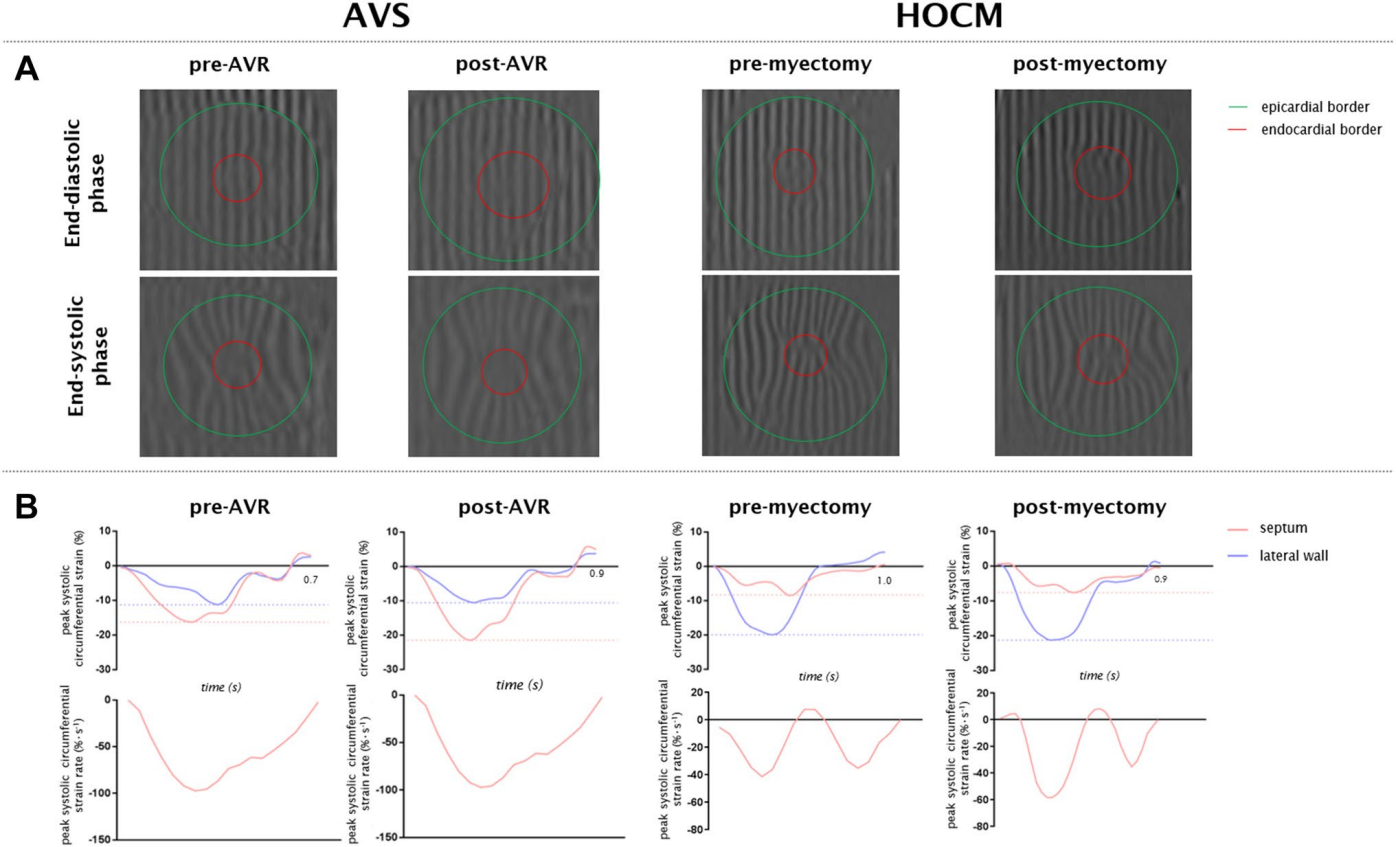
Representative tagging images of a patient with AVS and HOCM with corresponding strain signals. **a** At end-diastole and end-systole representative images are shown for a patient with AVS (left) and HOCM (right) before (pre) and after (post) surgical treatment. **b** Corresponding peak circumferential strain and systolic circumferential strain rate curves are presented

The cine images were analyzed off-line by a single investigator, using MASS analysis software (Medis medical imaging systems, v2.1, Leiden, The Netherlands). Endocardial contours were drawn at end-diastole and end-systole to calculate LV end-diastolic volume (LVEDV), LV end-systolic volume (LVESV), and LVEF. Addition of epicardial contours was used to calculate LV mass and wall thickening. Tissue tagging images were analyzed by Intag [20] software (CREATIS, Lyon, France) to quantify myocardial motion using the SinMod technique and estimate regional peak circumferential strain components (Lagrangian and systolic and diastolic strain rate, Fig. 1). Myocardial strain was measured in the mid myocardial layer which has been reported to be the most reproducible [21]. The software runs as a plug-in for OsiriX (v6.5, Pixmeo, Switzerland) [22]. Analysis of the LV was calculated according to the 17 segment AHA model [23]. To investigate regional effects after surgical treatment in both patient groups, strain analyses were derived from the septum (average of segment 8 and 9) and lateral wall (average of segment 11 and 12). Both areas were compared with each other. The lateral wall served as a remote area in which no regional treatment was performed. Because of the lack of tissue tagging on the long axis, global and regional longitudinal strain was analyzed off-line using CVi 42 software (Circle Cardiovascular Imaging, Calgery, Canada). Semi-automatic endo- and epicardial contours were drawn on the four, three and two chamber cine images. To compare septal and lateral longitudinal strain, the mean of the four septal segments of basal antero- and inferoseptal segments and four lateral segments of basal and mid antero- and inferolateral segments were calculated.

Statistical analysis was performed using SPSS software (version 22.0; SPSS, Chicago, IL, USA). All variables were visually checked for normal distribution by appreciation of the histogram with separate needs of the patient groups. All data were not normally distributed and presented by median with interquartile range and was compared to healthy controls with a Mann-Whitney-U test. Measurements prior to, and after surgical therapy were compared within groups using Wilcoxon signed-rank test. Exact Chi square test was used for categorical variables. As three separate statistical tests were performed for each outcome measure in each group of patients (comparison of pre- and post-measurement and separate comparison of pre- and post- measurements to the control group) we used a two-sided significance level of 0.05/3 to account for multiple testing.

## Results

### Myocardial function in aortic valve stenosis before surgery

**Fig. 2:**
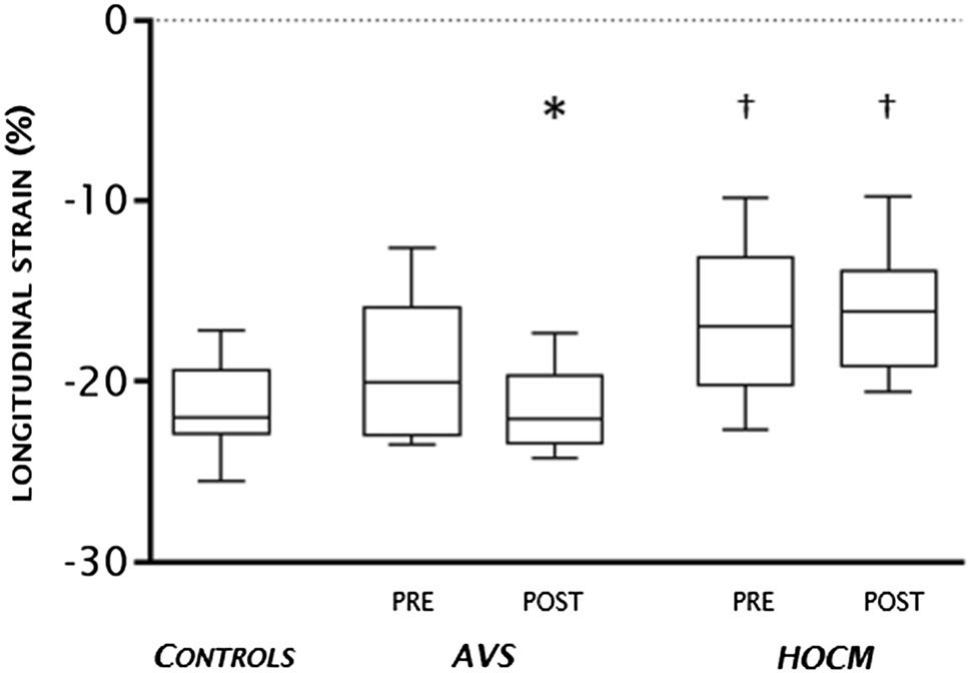
Global longitudinal strain. Global longitudinal strain is depicted for AVS before (pre) and after (post) surgery, HOCM before (pre) and after (post) surgery and healthy controls. AVS patients showed similar longitudinal strain compared with healthy controls both before and after AVR therapy. HOCM patients revealed a significantly lower longitudinal strain compared with healthy controls even after myecomy. †*p* < 0.01 versus controls. *p < 0.05 follow up versus baseline. All data is presented by median with interquartile range. *AVS * aortic valve stenosis; *HOCM  * hypertrophic obstructive cardiomyopathy

In the AVS group none of the patients used betablockers, one patient used an ACE inhibitor and four patients used a statin before surgery. Pressure gradients were significantly higher in AVS with a concomitant higher LA volume, as presented in Tables 1 and 2. End-diastolic wall thickness (EDWT) was significantly higher compared with controls both in the septum and the lateral wall (*p* < 0.001). Except for LV mass, all global LV dimensions were comparable to controls (Table 2). Septal and lateral wall thickening were significantly lower compared with controls (Table 3, *p* < 0.001), whereas only septal circumferential strain was significantly reduced (Table 3, *p* = 0.013). Septal systolic strain rate was reduced compared with controls (− 34 [− 44, − 27] vs. − 47 [− 58, − 34] % s^−1^, *p* = 0.005). Lateral systolic and diastolic strain rates before AVR were similar to controls (Table 3). Global longitudinal strain was similar to healthy subjects (Fig. 2).

**Table 1:** Baseline characteristics of controls, aortic valve stenosis and hypertrophic cardiomyopathy patients

	Controls (n = 14)	HOCM pre-myectomy (n = 10)	AVS pre-AVR (n = 10)	*p*-value HOCM pre-myectomy versus controls	*p*-value AVS pre-AVR versus controls	*p*-value HOCM pre-myectomy versus AVS pre-AVR
Age (years)	50 [40, 57]	53 [47, 65]	59 [54, 68]	0.37	0.009	0.14
Gender (male/female)	9/5	6/4	7/3	0.83	0.77	0.64
SBP (mmHg)	119 [112, 134]	106 [103, 123]	123 [107, 130]	0.04	0.55	0.06
DBP (mmHg)	71 [65, 75]	59 [57, 70]	72 [60, 78]	0.03	0.89	0.22
MAP (mmHg)	88 [81, 94]	76 [71, 88]	89 [76, 95]	0.03	0.71	0.14
Peak gradient Ao/LVOT (mmHg)*	NA	26 [15, 54]	85 [72, 107]	NA	NA	0.001
Mean gradient Ao/LVOT (mmHg)*	NA	14 [8, 23]	49 [42, 62]	NA	NA	< 0.001
Heart rate (/min)	69 [63, 78]	59 [57, 61]	68 [63, 70]	0.003	0.403	0.005

Data is presented as median and interquartile range

*Ao* aortic, *AVS* aortic valve stenosis, *DBP* diastolic blood pressure, *HOCM* hypertrophic obstructive cardiomyopathy, *LVOT* left ventricular outflow tract, *MAP* mean arterial pressure, *NA* not applicable, *POST* after surgical therapy, *PRE* before surgical therapy, *SBP* systolic blood pressure

*Measured by Doppler echocardiography

**Table 2:** Aortic valve stenosis before (PRE) and after (POST) aortic valve replacement: global characteristics

	Controls (n = 14)	AVS pre-AVR (n = 10)	*p*-value controls versus AVS pre-AVR	AVS post-AVR (n = 10)	*p*-value AVS pre-AVR versus AVS post-AVR	*p*-value AVS post-AVR versus controls
Global LV characteristics						
LV EDV (ml m^−2^)	90 [81, 104]	101 [84, 118]	0.29	84 [74, 102]	0.01	0.59
LV ESV (ml m^−2^)	36 [26, 40]	40 [29, 55]	0.29	31 [26, 39]	0.01	0.55
LVEF (%)	61 [57, 66]	59 [52, 64]	0.37	63 [59, 66]	0.29	0.67
SV (ml)	114 [101, 124]	111 [97, 128]	0.89	106 [86, 127]	0.17	0.63
CO (L min^−1^)	7.9 [6.5, 8.8]	8.0 [7, 10.1]	0.47	7.1 [6.2, 8.2]	0.01	0.29
LVM (g m^−2^)	50 [44, 54]	94 [79, 119]	< 0.001	72 [59, 89]	0.005	< 0.001
LGE mass (%) LV	0	0 [0, 1.4]	NA	0 [0, 1.4]	0.99	NA
LA characteristics						
LA volume (ml m^−2^)	45 [42, 50]	60 [53, 66]	< 0.001	48 [40, 57]	0.005	0.55
LAEF (%)	57 [54, 29]	53 [47, 58]	0.15	55 [48, 59]	0.24	0.37
Pressure gradients						
Peak gradient Ao (mmHg)*	NA	85 [72, 107]	NA	23 [14, 32]	0.005	NA
Mean gradient Ao (mmHg)*	NA	49 [42, 62]	NA	11 [7, 15]	0.005	NA
Cardiopulmonary exercise test						
Peak VO2 (L min^−1^)	NA	1.98 [1.36, 2.61]	NA	2.21 [1.52, 2.74]	0.06	NA
Exercise capacity (Watt)	NA	155 [89, 216]	NA	161 [97, 224]	0.008	NA

Data is presented as median (interquartile range)

*CO* cardiac output, *EDV* end-diastolic volume, *ESV* end-systolic volume, *AVR* aortic valve replacement, *AVS* aortic valve stenosis, *LGE* late gadolinium enhancement, *LVM* LV mass, *LAEF* left atrial emptying fraction, *LVEF* LV ejection fraction, *POST* after surgical therapy, *PRE* before surgical therapy, *SV* stroke volume, *VO2* oxygen consumption

*Measured by Doppler echocardiography

**Table 3:** Aortic valve stenosis before (PRE) and after (POST) aortic valve replacement: regional characteristics

		Controls (n = 14)	AVS pre-AVR (n = 10)	*p*-value controls versus AVS pre-AVR	AVS post-AVR (n = 10)	*p*-value AVS pre-AVR versus AVS post-AVR	*p*-value AVS post-AVR versus controls
Septal wall	ED wall thickness (mm)	6 [6, 7]	11 [11, 13]	< 0.001	9 [8, 10]	0.005	< 0.001
	Wall thickening (%)	80 [64, 97]	37 [33, 59]	0.001	54 [43, 74]	0.10	0.013
	Circumferential strain						
	Peak circumferential strain (%)	− 17 [− 18, − 14]	− 14 [− 16,− 12]	0.013	− 16 [− 18, − 13]	0.11	0.37
	Peak systolic circumferential strain rate (% s^−1^)	− 47 [− 58, − 43]	− 34 [− 44, − 27]	0.005	− 40 [− 53, − 32]	0.20	0.14
	Peak diastolic circumferential strain rate (% s^−1^)	36 [26, 39]	27 [12, 43]	0.29	26 [22, 42]	0.24^a^	0.57
Lateral wall	ED wall thickness (mm)	6 [5, 6]	10 [9, 11]	< 0.001	9 [8, 9]	0.01	< 0.001
	Wall thickening (%)	91 [77, 110]	31 [27, 52]	< 0.001	54 [38, 77]	0.01	0.01
	Circumferential strain						
	Peak circumferential strain (%)	− 17 [− 19,− 13]	− 16 [− 21,− 10]	0.84	− 16 [− 21,− 12]	0.88	0.98
	Peak systolic circumferential strain rate (% s^−1^)	− 37 [− 50,− 26]	− 50 [− 72, −30]	0.24	− 45 [− 51,− 38]	0.46	0.19
	Peak diastolic circumferential strain rate (% s^−1^)	13 [10, 18]	17 [13, 26]	0.13	18 [12, 25]	0.35^a^	0.19

Data is presented as median (interquartile range)

*AVR* aortic valve replacement, *AVS* aortic valve stenosis, *ED* end-diastolic, *POST* after surgical therapy, *PRE* before surgical therapy

^a^Due to tagfading two patients were excluded from lateral diastolic strain rate analysis

### Myocardial function in hypertrophic obstructive cardiomyopathy before surgery

In the HOCM group, eight patients used betablockers, one used an ACE inhibitor and one used a diuretic before myectomy. At baseline HOCM presented with a lower heart rate, probably due to the usage of betablockers (*p* = 0.003). Pressure gradients were significantly higher in HOCM with a concomitant higher LA volume, as listed in Tables 1 and 4. Regarding global myocardial function, HOCM patients had smaller LVESV, higher LVEF, and higher LV mass compared to controls (Table 4). Septal and lateral EDWT were higher than controls (Table 5, *p* < 0.001). Septal wall thickening and septal circumferential strain were significantly reduced compared with controls (Table 5, *p* < 0.01). Similar to AVS patients, septal systolic and diastolic strain rate were reduced compared with controls before myectomy (− 28 [− 44, − 17] vs. − 47 [− 58, − 34] % s^−1^, *p* = 0.005; 13 [9, 33] vs. 36 [26, 39] % s^−1^, *p* = 0.02, resp.). Lateral systolic and diastolic strain rate were comparable to controls (Table 5). Interestingly, there were no differences between systolic and diastolic strain rates between AVS and HOCM patients before surgery (for both *p* = 0.53). Global longitudinal strain was significantly affected compared with controls (− 16 [− 19, − 14] vs. − 22 [23, − 19] %, *p* = 0.007, Fig. 2).

**Table 4:** Hypertrophic obstructive cardiomyopathy before (PRE) versus after (POST) septal myectomy: global characteristics

	Controls (n = 14)	HOCM pre-myectomy (n = 10)	*p*-value controls vs HOCM pre-myectomy	HOCM post-myectomy (n = 8)	*p*-value HOCM pre-myectomy versus HOCM post-myectomy	* p* -value HOCM post-myectomy versus controls
Global LV characteristics						
LV EDV (ml m^−2^)	90 [81, 104]	93 [78, 104]	0.93	76 [67, 96]	0.16	0.21
LV ESV (ml m^−2^)	36 [26, 40]	24 [21, 31]	0.02	25 [20, 29]	0.67	0.24
LVEF (%)	61 [57, 66]	73 [65, 74]	0.009	66 [61, 73]	0.21	0.13
SV (ml)	114 [101, 124]	133 [92, 166]	0.34	112 [91, 146]	0.21	0.92
CO (L min^−1^)	7.9 [6.5, 8.8]	7.6 [2.8, 10]	0.55	8.4 [7.3, 9.5]	0.26	0.53
LVM (g m^−2^)	50 [44, 54]	92 [90, 114]	< 0.001	81 [67, 91]	0.03	< 0.001
LGE mass (%) LV	0	4.0 [1.7, 11.3]	NA	4.9 [1.1, 36.4]	0.88	NA
LA characteristics						
LA volume (ml m^−2^)	45 [42, 50]	84 [69, 114]	< 0.001	62 [51, 80]	0.012	< 0.001
LAEF (%)	57 [54, 29]	41 [27, 46]	< 0.001	46 [42, 54]	0.16	0.002
Pressure gradients						
Peak gradient LVOT (mmHg)*	NA	14 [8, 23]	NA	8 [5, 19]	0.02	NA
Mean gradient LVOT (mmHg)*	NA	26 [15, 54]	NA	5 [3, 9]	0.02	NA
Cardiopulmonary exercise test						
Peak VO2 (L min^−1^)	NA	1.89 [1.26, 2.05]	NA	1.92 [1.50, 2.79]	0.89	NA
Exercise capacity (Watt)	NA	140 [105, 170]	NA	152 [121, 221]	0.043	NA

Data is presented as median (interquartile range)

*HOCM* hypertrophic obstructive cardiomyopathy; other abbreviations as in Table 2

**Table 5:** Hypertrophic obstructive cardiomyopathy before (PRE) versus after (POST) septal myectomy: regional characteristics

		Controls (n = 14)	HOCM pre-myectomy (n = 10)	*p*-value controls versus HOCM pre-myectomy	HOCM post-myectomy (n = 8)	*p*-value HOCM pre-myectomy versus post-myectomy	*p*-value HOCM post-myectomy versus controls
Septal wall	ED wall thickness (mm)	6 [6, 7]	15 [13, 17]	< 0.001	13 [10, 16]	0.04	< 0.001
	Wall thickening (%)	80 [64, 97]	50 [33, 66]	0.009	61 [36, 63]	0.33	0.02
	Circumferential strain						
	Peak circumferential strain (%)	− 17 [− 18, − 14]	− 10 [− 15, − 9]	0.003	− 8 [− 10, − 7]	0.02	< 0.001
	Peak systolic circumferential strain rate (% s^−1^)	− 47 [− 58, − 43]	− 28 [− 44, − 17]	0.005	− 13 [− 17, − 6]	0.03	< 0.001
	Peak diastolic circumferential strain rate (% s^−1^)	36 [26, 39]	13 [9, 33]	0.02	15 [10, 23]	0.46a	0.002
Lateral wall	ED wall thickness (mm)	6 [5, 6]	10 [9, 12]	< 0.001	10 [8, 11]	0.78	< 0.001
	Wall thickening (%)	91 [77, 110]	83 [49, 128]	0.71	98 [60, 123]	0.26	0.97
	Circumferential strain						
	Peak circumferential strain (%)	− 17 [− 19, − 13]	− 16 [− 19, − 13]	0.78	− 19 [− 21, − 16]	0.17	0.22
	Peak systolic circumferential strain rate (% s^−1^)	− 37 [− 50, − 26]	− 46 [− 56, − 32]	0.48	− 53 [− 64, − 42]	0.23	0.046
	Peak diastolic circumferential strain rate (% s^−1^)	13 [10, 18]	15 [11, 22]	0.44	17 [11, 22]	0.92^a^	0.40

Data is presented as median (interquartile range)

*HOCM * hypertrophic cardiomyopathy; other abbreviations as in Table 4

^a^Due to tagfading two patients were excluded from diastolic strain rate analysis

### Effect on myocardial function after aortic valve replacement

Both peak and mean transvalvular pressure gradients in AVS patients significantly decreased after surgery (from 85 [72, 107] to 23 [14, 32] mmHg, and from 49 [42, 62] to 11 [7, 15] mmHg, *p* = 0.005, respectively). Global LV dimensions and LA volume decreased after AVR, however there was no significant change in LVEF and LAEF (Table 2). Lateral wall thickening significantly improved after AVR and septal wall thickening showed a trend towards improvement albeit non-significant compared to before AVR (Table 3; Fig. 1 Supplemental Material). Both global (− 14 [− 16, 13] to − 17 [− 18, − 14] %, *p* = 0.4) and regional circumferential strain did not significantly improve after surgery (Table 3). Global longitudinal strain improved after AVR therapy (− 20 [− 23, − 20] to − 22 [− 23, − 20] %, *p* = 0.013, Fig. 2), with no differences between septal and lateral segments (Table 1 Supplementary Material). The amount of LGE after surgery did not change. Nonetheless, exercise capacity during cardiopulmonary exercise test showed a significant improvement from 155 [89, 216] to 161 [97, 224] Watt, *p* = 0.008. Peak VO2 only showed a trend toward improvement (1.98 [1.36, 2.61] vs. 2.21 [1.52, 2.74] L min^−1^, *p* = 0.06).

### Effect on myocardial function after septal myectomy

**Fig. 3:**
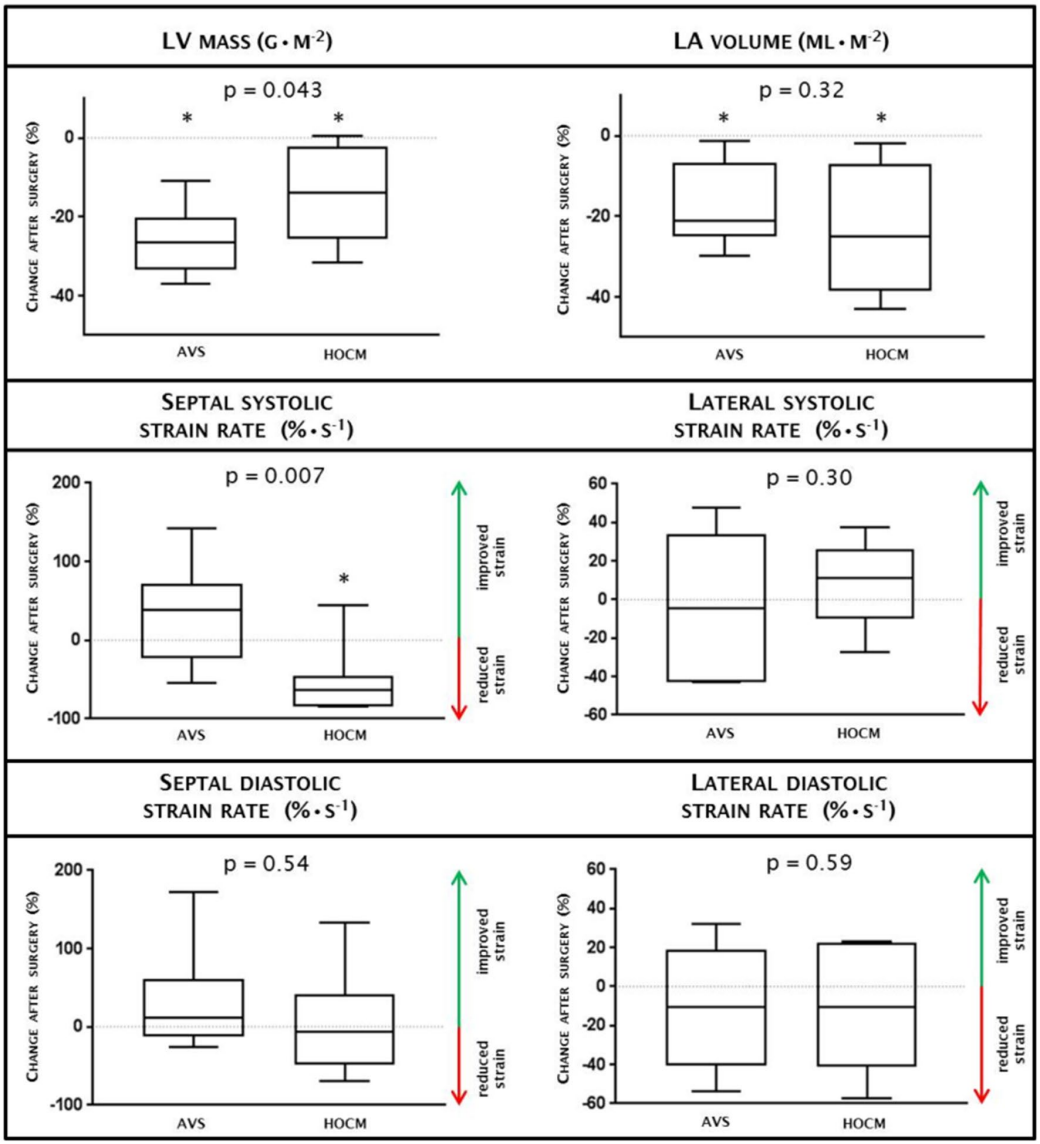
Change after surgery between AVS and HOCM. Change after surgery between AVS (n = 10) and HOCM (n = 8) are depicted for LV myocardial mass, left atrial volume, circumferential strain and regional systolic and diastolic stain rates. Values above zero in strain rates indicate improved strain, values below zero indicate reduced strain. An asterisk (*) indicates significant change within AVS or HOCM before vs after surgery (*p*-values are mentioned in Tables 2, 4). All data is presented by median with interquartile range. After surgery, HOCM demonstrates deterioration of septal systolic strain rate compared with AVS. Changes after surgery in regional diastolic strain rates were similar in HOCM compared to AVS. *AVS * aortic valve stenosis, *HOCM * hypertrophic obstructive cardiomyopathy, *LA * left atrial, *LV * left ventricular

From a total of ten HOCM patients, one patient declined follow up CMR, and another patient underwent pacemaker implantation due to an atrioventricular block. Measurements at baseline were included from ten patients, and at follow-up from the remaining eight patients. Both LVOT peak and mean gradients improved after septal myectomy (from 26 [15, 54] to 5 [3, 9] mmHg and 14 [8, 23] to 8 [5, 19] mmHg, *p* = 0.02, respectively). As was to be expected, septal EDWT decreased following myectomy. Also, LV mass and LA dimension decreased after myectomy, but without a change in LV dimensions, LVEF or LAEF (Table 4). Although wall thickening showed a non-significant difference after myectomy (Fig. 1 Supplementary Material), global circumferential strain did not improve after myectomy (− 13 [− 16, − 11] to − 14 [− 15, − 11] %, *p* = 0.52). With respect to regional circumferential strain only septal circumferential strain further deteriorated with worsening of septal systolic strain rate (Table 5). Additionally global and regional longitudinal strain did not improve after myectomy (Fig. 3; Table 1 Supplementary Material). The amount of late gadolinium enhancement showed no significant increase after myectomy. Similar to AVS, septal myectomy showed a significant increase in exercise capacity during cardiopulmonary exercise test (140 [105, 170] vs. 152 [121, 221] Watt, *p* = 0.043), while peak VO2 remained similar (1.89 [1.26, 2.05] vs. 1.92 [1.50, 2.79] L min^−1^, *p* = 0.89).

### Difference in myocardial adaptation after aortic valve replacement and septal myectomy

In both patient groups, there was a significant reduction in intraventricular pressure gradients with a concomitant decrease in LA volume (*p* < 0.01), suggesting lowering of diastolic filling pressures. Wall thickening improved after AVR, albeit without improvement in strain and strain rates. Similar to AVS patients, strain rates of the lateral wall were not affected after surgery for HOCM, while septal systolic circumferential strain rate further deteriorated, probably as a consequence of the myectomy. Both AVS and HOCM patients showed a reduction in LV wall thickness and mass after surgery, though this effect was more pronounced in AVS patients (*p* < 0.05, Fig. 3). Interestingly, both patient groups had an improvement in exercise capacity, most likely associated to the relieve in outflow obstruction.

## Discussion

This study provided the unique opportunity to prospectively investigate myocardial function in patients with two different etiologies in LVH, and to what extent reverse remodeling occurs in AVS and HOCM patients after surgical treatment, AVR and septal myectomy respectively. The main findings of the present study are that (1) both AVS and HOCM patients have significantly higher LV mass and LA volumes than controls, and in HOCM smaller LV volumes compared with AVS patients and controls; (2) both patient groups had lower septal wall thickening and systolic strain rates than controls; (3) both patient groups demonstrated reversed *structural* remodeling after surgery, with improved intraventricular pressure gradients and a concomitant decrease in LA volume, decreased wall thickness and LV mass; (4) only AVS patients showed *functional* improvement after surgical treatment, evident from improved global wall thickening but unaffected strain rates, whereas HOCM patients deteriorated with respect to septal wall thickening and systolic strain rate.

In both patient groups, intraventricular pressure gradients improved after surgery with a concomitant reduced LA volume, probably reflecting improved diastolic pressures. AVR is known to improve symptoms, reduce LV mass and wall thickness [24, 25], which is in line with the results in our cohort. Whereas Staron and colleagues demonstrated improvement in echocardiographic circumferential strain after AVR [26], in our cohort we found an even greater improvement comparable to normal values, suggesting that AVR reversibly affects the LV. A previous study using myocardial tagging showed global diastolic dysfunction before AVR and improvement after surgery [27]. This may be explained by the delayed and prolonged diastolic untwisting in AVS before AVR [28]. In this study AVS patients had normal diastolic function before AVR similar to controls, probably due to an earlier timing of the surgical intervention. While there is still debate to advise betablockers in order to reduce afterload in AVS patients [3], in our cohort none of the patients used betablockers. Taken together these findings confirm the benefit of decreasing the pressure gradient by valve replacement, thereby restoring structure and function and ultimately improving exercise capacity.

Whereas the increased pressure gradient in AVS is a static phenomenon, the pressure gradient in HOCM is of a dynamic nature, mainly depending on loading conditions [16]. Septal myectomy and alcohol ablation are both equally effective at reducing LVOT obstruction, however, septal myectomy is shown to be more effective in improving exercise parameters and results in a consistent septum reduction [29]. This study demonstrated a reduction in LV mass and septal wall thickness which is in line with previous results [30]. Although myectomy demonstrated reversed structural remodeling at the septum, surgery may have induced myocardial dysfunction. Global dysfunction of circumferential strain was also seen by Moravsky et al. by echocardiography [10], which is in agreement with our measurements by CMR. Regional analysis demonstrated impaired septal systolic and diastolic function before surgery, whereas the lateral wall showed similar values compared to healthy controls. The differences between septum and lateral wall may be explained by the difference of tissue characteristics. At the hypertrophied septum tissue characteristics demonstrate increased amount of extracellular volume and myocardial disarray compared with the lateral wall. These myocardial differences are in accordance with a longitudinal study in HOCM patients after alcohol septum ablation, in which septal systolic function further deteriorated after intervention [11]. Sommer et al. demonstrated reduced longitudinal strain up to 3 years after alcohol septal ablation in HOCM patients [31]. The current study also demonstrated reduced longitudinal strain even after myectomy, while contrast enhancement was similar compared to before myectomy [32]. In contrast to septal alcohol ablation therapy in HOCM patients, where a myocardial infarction is induced to achieve septal reduction which leads to increased scarring [11]. In our HOCM population scar is not responsible for the functional deterioration after myectomy. This implies that other mechanisms may be responsible for this loss in function, such as loss of myocyte integrity or progression of myocyte disarray [33]. In addition, the presence of sarcomere mutations continue to cause inhomogeneous contraction of the sarcomeres in the remaining cardiomyocytes after septal myectomy and consequently further reduce myocardial function. Although myectomy did not improve regional function in our population, a recent study demonstrated that myectomy in HOCM patients had a positive effect on the incidence of sudden cardiac death and implantable cardioverter-defibrillator discharge [34]. However, to be able to better understand and define the pathological process of functional loss of the myocardium, future studies using new imaging techniques, such as extracellular volume fraction assessment using T1 mapping, may be useful [35].

Diastolic dysfunction is a hallmark of HOCM, and seems to be largely caused by increased interstitial fibrosis leading to reduced LV compliance [36]. The reduction in LA volume after myectomy in our population suggests an improvement in diastolic intraventricular pressures, this study however did not demonstrate improvement in global or regional diastolic function, which might be explained by the reduced LV compliance [37]. Accordingly, an echocardiographic study also revealed reduction in LA volume without improvement in diastolic function and might be related to the disease history and increased development of interstitial fibrosis [12]. Summarizing, our findings we demonstrate reversed structural remodeling after myectomy, and worsening of functional remodeling at the septum. Even though functional remodeling deteriorated after surgery, HOCM patients managed to improve exercise capacity, which seems to be a direct result of relieving the LVOT obstruction by myectomy [29].

The present study demonstrates reversed structural remodeling in both AVS and HOCM patients after surgery, however, recovery in systolic function was only seen in AVS. Furthermore, HOCM patients demonstrated systolic functional deterioration which seems to be inherent to septal myectomy and the ongoing and irreversible cardiac pathophysiology. Although septal myectomy reduces the risk of sudden cardiac death, this study emphasizes the need for future research in therapies to enhance myocardial recovery.

However, there are several limitation in this study. Several medical centers were involved in the inclusion of patients for advanced imaging before and after cardiac surgery, and therefore, the included patients do not reflect the actual number of patients who yearly undergo cardiac surgery. As the number of participants included were limited, the conclusions should be interpreted carefully. Yet, even with this relative small study population we were able to demonstrate differences in myocardial adaptation after surgery for AVS and HOCM. Although tissue tagging is a novel and accurate method to assess myocardial function, tagline fading in end-diastole occurred in two patients who were therefore excluded from the diastolic strain rate analysis. Accurate detection of diffuse fibrosis using T1 mapping might have increased our understanding of the absence of functional recovery in HOCM. Furthermore, evaluation by CMR was performed at 4 months after surgery which seems reasonable but not necessarily the optimal timing to capture full remodeling and functional recovery.

## Electronic supplementary material

Below is the link to the electronic supplementary material.

Supplementary material 1 (DOCX 448 KB)

## Abbreviations

AVS: Aortic valve stenosis
AVR: Aortic valve replacement
HOCM: Hypertrophic obstructive cardiomyopathy
LV: Left ventricular
LVH: Left ventricular hypertrophy
LVOT: Left ventricular outflow tract
CMR: Cardiovascular magnetic resonance
EDWT: End-diastolic wall thickness
bSSFP: Balanced steady-state free precession
