# Supplementary material for: Myocardial adaptation after surgical therapy differs for aortic valve stenosis and hypertrophic obstructive cardiomyopathy
Source: Int J Cardiovasc Imaging. 2019 Mar 1;35(6):1089–100. doi: 10.1007/s10554-019-01563-3 (PMC6534665; doi:10.1007/s10554-019-01563-3)
Supplement: Supplementary file 1 — Supplementary material 1 (DOCX 448 KB) [file 10554_2019_1563_MOESM1_ESM.docx]

**Myocardial adaptation after surgical therapy differs for aortic valve stenosis and hypertrophic obstructive cardiomyopathy**

Rahana Y. Parbhudayal, MD^a,b,j^, Ahmet Güçlü, MD, PhD^c^, Alwin Zweerink, MD^a^, P. Stefan Biesbroek, MD^a^, Pierre Croisille, MD^d^, Patrick Clarysse, PhD^e^, Michelle Michels, MD, PhD^f^, Wim Stooker, MD, PhD^g^, Alexander B.A. Vonk, MD, PhD^h^, Peter M. van der Ven, PhD^i^, Albert C. van Rossum, MD, PhD^a^, Jolanda van der Velden, PhD^b,j^, Robin Nijveldt, MD, PhD^a^

^a^Department of Cardiology, ^b^Department of Physiology, Amsterdam UMC, Vrije Universiteit Amsterdam, Amsterdam Cardiovascular Sciences, The Netherlands

^c^Department of Cardiology, Isala Klinieken, Zwolle, The Netherlands

^d^Univ Lyon, UJM-Saint-Etienne, INSA, CNRS UMR 5520, Inserm U1206, Creatis, F-42023, Sint-Etienne, France

^e^Univ Lyon, INSA‐Lyon, Université Claude Bernard Lyon 1, UJM-Saint Etienne, CNRS, Inserm, Creatis UMR 5220, U1206, F‐69621, Lyon, France

^f^Department of Cardiology, Erasmus Medical Center, Rotterdam, The Netherlands

^g^Department of Cardiothoracic Surgery, Onze Lieve Vrouwe Gasthuis, Amsterdam, The Netherlands

^h^Department of Cardiothoracic Surgery, VU University Medical Center Amsterdam, The Netherlands

^i^Department of Epidemiology and Biostatistics, VU University Medical Center, Amsterdam, The Netherlands

^j^The Netherlands Heart Institute, Utrecht, The Netherlands

**The International Journal of Cardiovascular Imaging**

**Corresponding author:**

Robin Nijveldt, MD, PhD

Amsterdam UMC, Vrije Universiteit Amsterdam

Department of Cardiology

De Boelelaan 1117, 1081 HV, Amsterdam, The Netherlands

Fax: +31(0)20-444 33 95

Tel: +31(0)20-444 22 44

Email: [robin@nijveldt.net](mailto:robin@nijveldt.net)

**SUPPLEMENTAL MATERIAL**

***Supplemental Methods***

***CMR protocol***

A retrospectively gated balanced steady-state free-precession (bSSFP) cine-CMR short axis stack was acquired for the assessment of LV volumes, function and mass with the following imaging parameters: were 5-mm slice thickness with 5-mm gap between short-axis slices, temporal resolution <50 ms, repetition time 3.2 ms, echo time 1.54 ms, flip angle 70°, and typical image resolution 1.3 × 1.6 mm. A stack of 10 to 12 short-axis slices was used to cover the LV, as described previously [1].

***Myocardial tagging***

A multiple breath-hold, retrospective triggered bSSFP myocardial sinusoidal complementary tagged (CSPAMM) images were acquired to create non-invasive markers (tags) within the myocardium [2], with the following imaging parameters: temporal resolution was 14 ms. The imaging parameters were as follows: field of view: 300 × 300 mm2, flip-angle: 20°, repetition time: 3.6 ms, echo time: 1.8 ms, receiver bandwidth: 850 Hz/pixel, matrix size: 256 × 78, slice thickness: 6 mm, tag-line distance: 7 mm.

***Late gadolinium enhancement***

Late gadolinium enhancement (LGE) images were acquired 10-15 minutes after intravenous administration of 0.2 mmol∙kg-1 gadolinium for quantification of myocardial fibrosis [3]. None of the patients presented with a pattern of gadolinium enhancement similar to what is seen in amyloidosis.

**Results**

**Figure S1. Regional wall thickening**

**
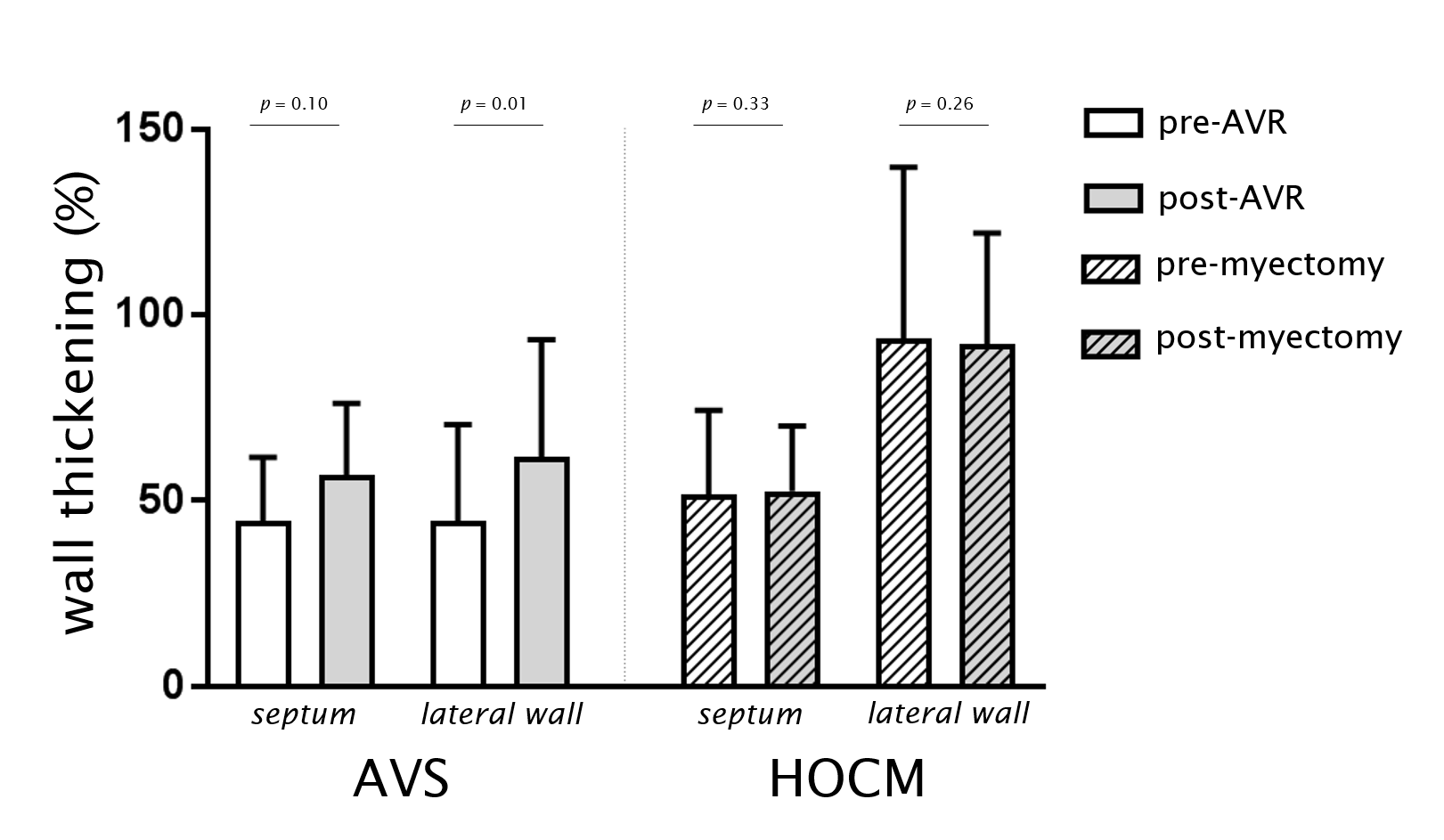
**

Wall thickening is depicted for AVS and HOCM patients at the septal and lateral wall before (pre) and after (post) surgery. Data is presented as median with range. AVS = aortic valve stenosis; HOCM = hypertrophic obstructive cardiomyopathy.

**Table S1. Regional longitudinal strain**

|  | Controls (n=14) | AVS pre-AVR (n=10) | p-value controls vs AVS | AVS post-AVR (n=10) | p-value  AVS pre-AVR vs AVS post-AVR | p-value  AVS post-AVR vs controls |
| --- | --- | --- | --- | --- | --- | --- |
| Septal | -19.5 [-22, -18] | -19 [-22, -18] | 0.9 | -21 [-22, -18] | 0.6 | 0.5 |
| Lateral | -21.8 [-25, -20] | -22 [-24, -15] | 0.8 | -25 [-26, -22] | 0.09 | 0.2 |

Data are presented as median (interquartile range). AVS = aortic valve stenosis; POST = after surgical therapy; PRE = before surgical therapy. Septal segments are the mean the four of basal and mid antero- and inferoseptal segments.

|  | Controls (n=14) | HOCM pre-myectomy (n=10) | p-value  controls vs HOCM pre-myectomy | HOCM post-myectomy (n=8) | p-value  HOCM pre-myectomy vs HOCM post-myectomy | p-value  HOCM post-myectomy vs controls |
| --- | --- | --- | --- | --- | --- | --- |
| Septal | -19.5 [-22, -18] | -17 [-20, -12] | 0.07 | -17 [-19, -9] | 0.7 | 0.01 |
| Lateral | -21.8 [-25, -20] | -19 [-24, -14] | 0.09 | -19 [-23, -15] | 0.6 | 0.06 |

Data are presented as median (interquartile range). HOCM = hypertrophic obstructive cardiomyopathy; POST = after surgical therapy; PRE = before surgical therapy. Septal segments are the mean the four of basal and mid antero- and inferoseptal segments.

**Supplemental References**

1 Marcus JT, Gotte MJ, DeWaal LK et al (1999) The influence of through-plane motion on left ventricular volumes measured by magnetic resonance imaging: implications for image acquisition and analysis. J Cardiovasc Magn Reson 1:1-6

2 Zwanenburg JJ, Kuijer JP, Marcus JT, Heethaar RM (2003) Steady-state free precession with myocardial tagging: CSPAMM in a single breathhold. Magn Reson Med 49:722-730

3 Bondarenko O, Beek AM, Hofman MB et al (2005) Standardizing the definition of hyperenhancement in the quantitative assessment of infarct size and myocardial viability using delayed contrast-enhanced CMR. J Cardiovasc Magn Reson 7:481-485
